# Development and multi-cohort validation of a clinical score for predicting type 2 diabetes mellitus

**DOI:** 10.1371/journal.pone.0218933

**Published:** 2019-10-09

**Authors:** Vanessa Kraege, Peter Vollenweider, Gérard Waeber, Stephen J. Sharp, Maite Vallejo, Oscar Infante, Mohammad Reza Mirjalili, Fatemeh Ezoddini-Ardakani, Hassan Mozaffari-Khosravi, Mohammad Hasan Lotfi, Masoud Mirzaei, Marie Méan, Pedro Marques-Vidal

**Affiliations:** 1 Department of Medicine, Internal Medicine, Lausanne University Hospital, Lausanne, Switzerland; 2 MRC Epidemiology Unit, University of Cambridge School of Clinical Medicine, Institute of Metabolic Science, Cambridge Biomedical Campus, Cambridge, England, United Kingdom; 3 Tlalpan 2020 Study, Department of Socio-Medical Research, National Institute of Cardiology, Ignacio Chávez, Mexico City, Mexico; 4 Shahid Sadoughi University of Medical Sciences, Yazd, Iran; Shanghai Diabetes Institute, CHINA

## Abstract

**Background and aims:**

Many countries lack resources to identify patients at risk of developing Type 2 diabetes mellitus (diabetes). We aimed to develop and validate a diabetes risk score based on easily accessible clinical data.

**Methods:**

Prospective study including 5277 participants (55.0% women, 51.8±10.5 years) free of diabetes at baseline. Comparison with two other published diabetes risk scores (Balkau and Kahn clinical, respectively 5 and 8 variables) and validation on three cohorts (Europe, Iran and Mexico) was performed.

**Results:**

After a mean follow-up of 10.9 years, 405 participants (7.7%) developed diabetes. Our score was based on age, gender, waist circumference, diabetes family history, hypertension and physical activity. The area under the curve (AUC) was 0.772 for our score, vs. 0.748 (p<0.001) and 0.774 (p = 0.668) for the other two. Using a 13-point threshold, sensitivity, specificity, positive and negative predictive values (95% CI) of our score were 60.5 (55.5–65.3), 77.1 (75.8–78.2), 18.0 (16.0–20.1) and 95.9 (95.2–96.5) percent, respectively. Our score performed equally well or better than the other two in the Iranian [AUC 0.542 vs. 0.564 (p = 0.476) and 0.513 (p = 0.300)] and Mexican [AUC 0.791 vs. 0.672 (p<0.001) and 0.778 (p = 0.575)] cohorts. In the European cohort, it performed similarly to the Balkau score but worse than the Kahn clinical [AUC 0.788 vs. 0.793 (p = 0.091) and 0.816 (p<0.001)]. Diagnostic capacity of our score was better than the Balkau score and comparable to the Kahn clinical one.

**Conclusion:**

Our clinically-based score shows encouraging results compared to other scores and can be used in populations with differing diabetes prevalence.

## Introduction

Diabetes mellitus is an important cause of morbidity, mortality and costs [1]. According to the NCD Risk factor Collaboration, the number of adults with diabetes worldwide increased from 108 million in 1980 to 422 million in 2014 [1]. Most new cases of diabetes will occur in low and middle-income countries, mainly due to the escalating prevalence of adiposity, rapidly changing dietary and physical activity behaviors, and lack of or late identification of people at risk of diabetes. Indeed, in low-income countries such as India, the prevalence of diabetes could be as high as 19.9% [2].

Early identification of people at risk of diabetes is paramount for adequate prevention by changes in lifestyle, and, if necessary, complemented by treatment. Thus, it is important to have an easily obtainable, inexpensive and reliable diabetes risk score. A review conducted in 2011 [3] identified as many as 145 diabetes risk models or scores and suggested that this number increases monthly. Among the 94 risk prediction models studied, 40 were based on biological variables [3]. Inclusion of biological variables in diabetes risk scores has a dual effect: it slightly improves the scores’ performances [4], but it also increases costs (staff and laboratory) and time (blood sampling, waiting for results). In a previous study, we have shown that the use of a diabetes risk score including biological variables cost an additional US $ 12.02 per patient compared to a score based on clinical data only [5]. Hence, in countries most affected by the current diabetes epidemic, the use of diabetes risk scores including biological variables may not be possible due to financial or laboratory constraints.

Thus, our first aim was to derive a diabetes risk score based solely on easily obtainable clinical data, and to validate it in a similar European population by comparing it with existing clinical scores. Our second aim was to examine the validity of our score regarding clinical utility and application on populations with different diabetes and obesity prevalence, and lesser economical means, using two different cohorts from Iran and Mexico.

## Materials and methods

### Sampling procedure

The CoLaus/PsyCoLaus study is a prospective population-based study intended to evaluate the prevalence and determinants of cardiovascular disease in the population of Lausanne, Switzerland. Details of the sampling procedure have been previously documented [6] and can be accessed online (www.colaus-psycolaus.ch). The source population was all adults aged 35 to 75 years in the Lausanne population register. A simple non-stratified random sample of 35% of the source population was drawn and an invitation letter sent. If the latter went unanswered, a second letter was sent and, if unanswered, several phone calls were performed. Recruitment began in June 2003 and ended in May 2006, enrolling 6733 participants who underwent an interview, a physical exam, and a blood analysis. The first follow-up was performed between April 2009 and September 2012, a mean of 5.6 years after the collection of baseline data. The second follow-up was performed between May 2014 and July 2016, a mean of 10.9 years after the collection of baseline data.

### Clinical and biological data

All participants were examined in the morning after a fast of at least 8 hours. They were probed about their personal and family history of cardiovascular disease and cardiovascular risk factors. All prescribed and over-the-counter medicines were collected via questionnaire. Smoking status was categorized as never, former (irrespective of the time since quitting) and current (irrespective of the amount smoked). Educational level was categorized as low (primary), middle (apprenticeship), upper middle (high school), and high (university) for highest completed level of education. Physical activity was defined by exercising at least twice per week for at least 20 minutes per session.

Body weight and height were measured with participants barefoot and in light indoor clothes. Body weight was measured in kilograms to the nearest 100 g using a Seca scale (Hamburg, Germany). Height was measured to the nearest 5 mm using a Seca (Hamburg, Germany) height gauge. Waist circumference was measured mid-way between the lowest rib and the iliac crest using a non-stretchable tape and the mean of two measurements was taken. Blood pressure (BP) was measured using an Omron HEM-907 automated oscillometric sphygmomanometer after at least a 10-minute rest in a seated position, and the mean of the last two measurements was used. Hypertension was defined by a SBP ≥130 mm Hg or a DBP ≥85 mm Hg or presence of antihypertensive drug treatment. Based on the review by Noble et al., the 130/85 mmHg threshold was preferred to the 140/90 mmHg one. High resting heart rate was defined by ≥ 68 beats per minute in men and ≥70 beats per minute in women.

Venous blood samples (50 mL) were drawn in the fasting state. Most biological assays were performed at the clinical laboratory of the Lausanne university hospital within 2 hours of blood collection. Glucose was assessed by glucose dehydrogenase with a maximum inter- and intra-assay CV of 2.1% and 1.0%, respectively; glycated hemoglobin (HbA1c) was assessed by HPLC (Bio-Rad, D-10TM, Reinach, Switzerland); total cholesterol by CHOD-PAP (1.6%-1.7%); HDL-cholesterol by CHOD-PAP + PEG + cyclodextrin (3.6%-0.9%); triglycerides by GPO-PAP (2.9%-1.5%).

### Incident diabetes mellitus

Two definitions of incident diabetes were used: 1) fasting glucose level ≥7 mmol/L and/or presence of an oral antidiabetic or insulin treatment, and 2) an HbA1c≥6.5% (48 mmol/mol) and/or presence of an oral antidiabetic or insulin treatment. As HbA1c was assessed only in the last follow-up, analyses were restricted to participants who attended the second follow-up.

### Other clinically-based diabetes mellitus risk scores for comparison

Two diabetes risk scores based solely on clinical data were considered: the score by Balkau et al. [7] derived from a French population and the clinical score by Kahn et al. [8] derived from a United States population (S1 Table). The score by Balkau et al. is based on five variables, and the score by Kahn et al. is based on eight variables. Both scores had been tested previously in our cohort [5].

### Inclusion and exclusion criteria

The original inclusion criteria into the CoLaus/PsyCoLaus Study were: 1) written informed consent; 2) willingness to take part in the examination and to provide blood samples; 3) French language ability. For this study, the following exclusion criteria were applied: 1) participants with type 1 and 2 diabetes at baseline; 2) no follow-up and 3) missing data for calculation of scores.

### Statistical analysis

Statistical analyses were conducted using Stata version 15.1 for Windows (Stata Corp, College Station, Texas, USA). Participants characteristics were expressed as number (percentage) for categorical variables or as mean±standard deviation for continuous variables. Between-group comparisons were performed using chi-square or Fisher’s exact test for categorical variables and student’s t-test for continuous variables. Multivariate analysis was conducted using logistic regression; to facilitate future scoring, all continuous variables (i.e. age, BMI, waist…) were categorized. Goodness of fit was assessed using the Hosmer-Lemeshow test with 10 categories; model quality was estimated using the Akaike and the Bayesian information criteria (AIC and BIC, respectively). For anthropometric data, models including only one of each parameter were computed, and the parameter providing the highest percentage of variance explained (pseudo-R^2^) was selected. The diabetes risk score was created taking into account the contribution of each variable significantly associated with diabetes in the logistic model. The score was built as the sum of assigned points, defined as the OR rounded to the nearest integer, as performed in another setting [9]. The best threshold to define a high risk of diabetes was based after visual examination of the graphs displaying the values of sensitivity, specificity, positive and negative predictive values according to the score values, priority being given to a high specificity and a high negative predictive value.

The diagnostic capacity of the different scores was assessed by the AUC [area under the ROC (receiver operating characteristic) curve] and corresponding 95% confidence intervals (CI). Comparisons between scores were performed using the **roccomp** command of Stata. Sensitivity, specificity, positive and negative predictive values and their corresponding 95% CIs were computed using incident diabetes (definition 1) as gold standard. The number needed to screen (NNS) to detect one case of diabetes was computed as the number of detected diabetes cases (i.e. true positives) divided by the total number of participants screened. Statistical significance was assessed for a two-sided test with p<0.05.

As women with gestational diabetes are at higher risk of developing diabetes type 2, a last sensitivity analysis was performed after excluding women with personal history of gestational diabetes.

### External validation cohorts

The performance of our diabetes risk score relative to two other clinically based diabetes risk scores was assessed in three cohorts. The European cohort included data from four countries (France, Germany, Netherlands and UK) of the EPIC-Europe cohort study [10]; these four countries were included because all relevant variables were available. Incident diabetes was defined using multiple sources of evidence including self-report, linkage to primary-care registers, secondary-care registers, medication use, hospital admissions and mortality data [11]. Tlalpan 2020 is a cohort of participating Mexico City residents recruited through promotional strategies in Mexico City, Mexico [12]; incident diabetes was defined as a fasting plasma glucose ≥7 mmol/L. The Shahedieh cohort study included data from a population-based survey in the province of Yazd, Iran [13] where incident diabetes was defined as a fasting plasma glucose ≥7 mmol/L.

We report our results according to the TRIPOD (transparent reporting of a multivariable prediction model for individual prognosis or diagnosis) statement [14].

### Ethical considerations

The institutional Ethics Committee of the University of Lausanne, which afterwards became the Ethics Commission of Canton Vaud (http://www.cer-vd.ch) approved the baseline CoLaus/PsyCoLaus study (reference 16/03, decisions of January 13 and February 10, 2003); the approval was renewed for the first (reference 33/09, decision of February 23, 2009) and the second (reference 26/14, decision of March 11, 2014) follow-up.

The institutional review boards for the various EPIC cohorts are the following: United Kingdom: Norfolk Research Ethics Committee; France: CNIL (Commission nationale de l’informatique et des libertés); Netherlands: Bilthoven Medical Ethical Committee of TNO Nutrition and Food Research; Institutional Review Board of the University Medical Center Utrecht; Germany: Ethical committee of the State of Brandenburg, Germany.

The Institutional Bioethics Committee of the INCICh, within the ethical framework of the seventh revision of the Declaration of Helsinki and the Regulations of the General Health Law in Matters of Research for Health of Mexico, approved the Tlalpan 2020 study of the incidence of systemic hypertension in a cohort of Mexico City residents and the written consent formats, under number 13–802 on February 19, 2013; since then, protocol and writer consent had been renewed yearly (September 23, 2014; November 17, 2015; September 13, 2016; May 16, 2017; September 26, 2018).

The institutional Ethics Committee of Digestive Disease Research Institute at Tehran University of Medical Sciences (http://ethics.research.ac.ir/) approved the baseline Shahedieh cohort study (reference: IR.TUMS.DDRI.Rec.1396.1); the approval was renewed for the first follow-up (reference IR.SSU. Rec.1397.135, decision of January 22, 2019).

All studies were performed in agreement with the Helsinki declaration and its former amendments. All participants gave their signed informed consent before entering the studies.

## Results

### Characteristics of participants

**Fig 1:**
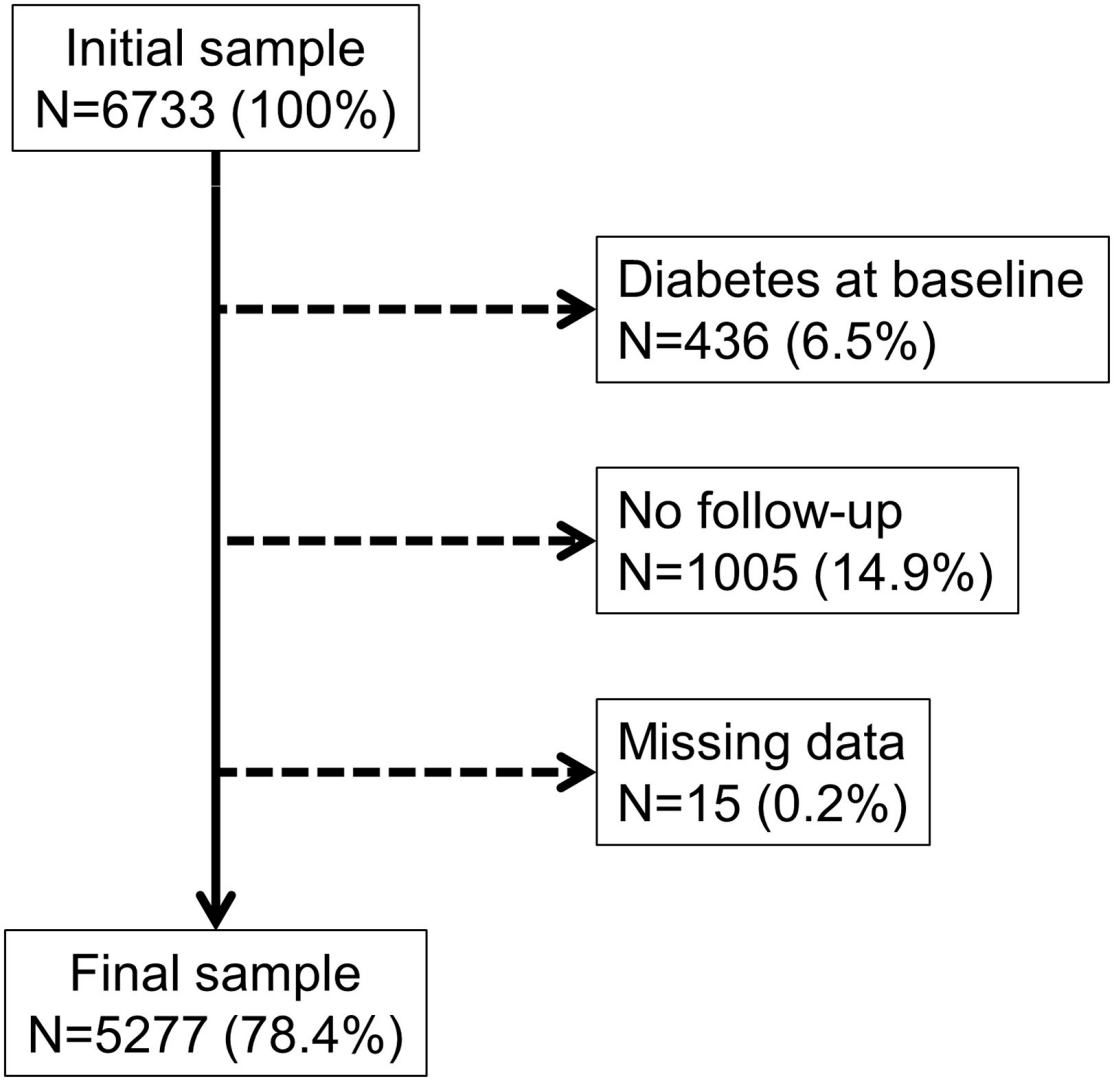
Flowchart of the participants of the CoLaus/PsyCoLaus study.

Of the initial 6733 patients, 21.6% were excluded, leaving 5277 participants (78.4%) for analysis. The reasons for exclusion are summarized in Fig 1 and the comparison between excluded and included participants is provided in S2 Table. Excluded participants were older, with higher BMI and waist circumference. They were also more frequently men, sedentary, of lower educational level, former or current smokers, with higher alcohol consumption, with a personal history of CVD, hypertension and lipid lowering drugs, and with a parental or family history of diabetes.

### Incidence of diabetes and score components

Between baseline and second follow-up, 405 participants (7.7%) developed diabetes as based on fasting plasma glucose. The bivariate comparison of 19 candidate variables between participants who developed and who remained free of diabetes is summarized in Table 1. In both genders, participants who developed diabetes were older, with higher BMI, waist, waist to height and waist to hip ratios. Participants who developed diabetes were also of lower educational level, had a higher frequency of hypertension, lipid lowering drugs, personal history of CVD and family history of diabetes, and a lower frequency of leisure-time physical activity (Table 1).

**Table 1:** Bivariate analysis of the factors associated with incident diabetes mellitus, stratified by gender. CoLaus/PsyCoLaus study, Lausanne, Switzerland, 2003–2017.

	Women			Men		
	Non-diabetic	Diabetic	P-value	Non-diabetic	Diabetic	P-value
Sample size	2755	149		2117	256	
Age (years)	52.2 ± 10.5	57.5 ± 9	<0.001	50.5 ± 10.3	54.9 ± 10.1	<0.001
Anthropometry						
Height (cm)	163 ± 7	162 ± 6	0.016	175 ± 7	174 ± 7	0.011
Weight (kg)	64.9 ± 11.7	75.9 ± 13.6	<0.001	79.7 ± 12	86.3 ± 13.2	<0.001
Body mass index (kg/cm^2^)	24.5 ± 4.3	29.1 ± 5.0	<0.001	25.9 ± 3.5	28.4 ± 3.9	<0.001
Waist circumference (cm)	81.6 ± 11.1	95.0 ± 12.5	<0.001	93.5 ± 10	100.9 ± 10.3	<0.001
Waist to height ratio	50.2 ± 7.2	58.9 ± 8.0	<0.001	53.4 ± 5.8	58.0 ± 6.0	<0.001
Waist to hip ratio	0.82 ± 0.07	0.88 ± 0.06	<0.001	0.92 ± 0.06	0.95 ± 0.05	<0.001
Educational level			0.001			<0.001
University	494 (17.9)	13 (8.7)		573 (27.1)	41 (16.0)	
High school	730 (26.5)	33 (22.2)		523 (24.7)	55 (21.5)	
Apprenticeship	969 (35.2)	57 (38.3)		735 (34.7)	106 (41.4)	
Mandatory education	562 (20.4)	46 (30.9)		286 (13.5)	54 (21.1)	
Smoking status			0.434			0.024
Never	1292 (46.9)	62 (41.6)		758 (35.8)	70 (27.3)	
Former	785 (28.5)	48 (32.2)		779 (36.8)	103 (40.2)	
Current	678 (24.6)	39 (26.2)		580 (27.4)	83 (32.4)	
BP ≥ 130/85 mm Hg	963 (35.0)	92 (61.7)	<0.001	1026 (48.5)	172 (67.2)	<0.001
Hypertension †	1063 (38.6)	101 (67.8)	<0.001	1100 (52.0)	193 (75.4)	<0.001
High resting heart rate ‡	1155 (41.9)	62 (41.6)	0.940	892 (42.1)	127 (49.6)	0.022
Lipid lowering drugs	216 (7.8)	26 (17.5)	<0.001	222 (10.5)	44 (17.2)	0.001
Alcohol ≥35 drinks/week	9 (0.3)	0 (0)	1.00 §	60 (2.8)	14 (5.5)	0.022
Caffeinated drinks (units/day)			0.644			0.197
None	178 (6.5)	12 (8.1)		128 (6.1)	13 (5.1)	
1–3	1819 (66.0)	94 (63.1)		1350 (63.8)	170 (66.4)	
4–6	654 (23.7)	35 (23.5)		533 (25.2)	54 (21.1)	
>6	104 (3.8)	8 (5.4)		106 (5.0)	19 (7.4)	
Leisure-time PA ≥2/week	1599 (58.0)	67 (45.0)	0.002	1162 (54.9)	114 (44.5)	0.002
Personal history of CVD	116 (4.2)	13 (8.7)	0.009	110 (5.2)	27 (10.6)	0.001
Parental history of diabetes			<0.001 §			0.015 §
No	2262 (82.1)	99 (66.4)		1777 (83.9)	195 (76.2)	
Mother only	262 (9.5)	26 (17.5)		164 (7.8)	31 (12.1)	
Father only	210 (7.6)	21 (14.1)		165 (7.8)	28 (10.9)	
Both	21 (0.8)	3 (2.0)		11 (0.5)	2 (0.8)	
Family history of diabetes						
Type 1+2	612 (22.2)	65 (43.6)	<0.001	402 (19.0)	72 (28.1)	0.001
Type 2	586 (21.3)	64 (43.0)	<0.001	390 (18.4)	71 (27.7)	<0.001

BP, blood pressure; CVD, cardiovascular disease; PA, physical activity.

^†^ defined by SBP≥130 mm Hg or DBP ≥ 85 mm Hg or presence of antihypertensive drug treatment;

^‡^, defined by ≥68 beats per minute in men and ≥70 beats per minute in women.

Results are expressed as mean ± standard deviation or as number of participants (%). Between-group comparisons performed using student’s t-test for continuous variables and chi-square or Fisher’s exact test (§) for categorical variables.

The variables significantly associated with incident diabetes on bivariate analysis were introduced in a multivariable logistic regression model. Based on the results of the logistic regression and the percentage of variance explained, the following variables were selected for the diabetes risk score: gender, age, waist circumference, hypertension, family history of diabetes, and physical activity. The scoring system is provided in Table 2. First, we developed a separate scoring system for men and women. Nevertheless, as we wanted to keep the score as simple as possible, we developed a final scoring, applicable to either sex. The odd ratios enabling the scores are provided in S3 Table. Waist was the most important determinant of incident diabetes, while no increasing effect of age categories were found. The threshold of 13 points provided the best combination of sensitivity and specificity.

**Table 2:** Clinical score for predicting type 2 diabetes mellitus risk.

	Men	Women	Final scoring
Male gender	-	-	2
Age group (years)			
[45–54]	2	3	2
[55–64]	2	3	2
[65–75]	2	3	2
Waist (cm)			
70–79 W, 80–89 M	3	2	2
80–89 W, 90–99 M	6	6	6
90–99 W, 100–109 M	12	12	12
100–109 W, 110–120 M	14	22	18
110+ W, 120+ M	32	31	32
Family history of diabetes §	2	2	2
Physical inactivity ǂ	1	1	1
Hypertension †	2	2	2

M, men; W, women.

^§^: father, mother or siblings;

^ǂ^: less than twice 20 minutes leisure physical activity per week;

^†^: systolic blood pressure ≥ 130 mm Hg and/or diastolic blood pressure ≥ 85 mmHg and/or antihypertensive drug treatment.

The comparison between our scoring system and the other diabetes risk scores for the CoLaus/PsyCoLaus study is provided in Table 3. Based on the AUC; our score performed better than the score by Balkau et al., while no differences were found with the score by Kahn et al. Similar findings were observed when the analysis was split by gender. The results of the diagnostic capacity of the different scores, overall and stratified by gender are provided in Table 4. Compared to the score by Balkau et al., our score had a higher sensitivity and negative predictive value, but a lower specificity. Compared to the score by Kahn et al., our score had comparable diagnostic capacities, and a slightly better specificity (Table 4). Similar findings were observed when the analysis was split by gender (Table 4). The goodness of fit and information criteria are provided in S4 Table.

**Table 3:** Areas under the receiver operating characteristic (ROC) curves and incidence of diabetes per quintile of diabetes risk estimation score, overall and stratified by gender, CoLaus/PsyCoLaus study, Lausanne, Switzerland, 2003–2017.

	AUC (95% CI)	P-value §	First	Second	Third	Fourth	Fifth
**All participants**							
CoLaus/PsyCoLaus	0.772 (0.750–0.794)		1.0	3.0	6.0	10.8	20.2
Balkau	0.748 (0.726–0.770)	<0.001	1.0	5.0	9.1	NA	19.7
Kahn clinic	0.774 (0.753–0.796)	0.668	0.7	2.5	5.4	10.7	20.0
**Women (n = 2904)**							
CoLaus/PsyCoLaus	0.806 (0.772–0.840)		0.8	2.4	4.3	7.2	18.4
Balkau	0.788 (0.753–0.822)	0.024	0.8	2.8	6.4	NA	16.9
Kahn clinic	0.807 (0.774–0.839)	0.948	0.4	1.5	4.6	7.6	17.8
**Men (n = 2373)**							
CoLaus/PsyCoLaus	0.719 (0.688–0.751)		1.8	3.9	7.3	13.6	21.5
Balkau	0.700 (0.670–0.730)	0.065	1.6	7.8	11.5	NA	22.2
Kahn clinic	0.728 (0.698–0.758)	0.283	1.5	4.2	6.4	13.9	21.4

Results are expressed as area under the curve and (95% confidence interval), and as percentage of participants developing diabetes during the 10.9 year follow-up. Diabetes was defined as fasting glucose level ≥7 mmol/L and/or presence of an oral antidiabetic or insulin treatment

**Table 4:** Diagnostic capacity of the different scores, overall and stratified by gender, CoLaus/PsyCoLaus study, Lausanne, Switzerland, 2003–2017.

	Threshold	Sensitivity	Specificity	Positive PV	Negative PV	N needed to screen
**All participants**						
CoLaus/PsyCoLaus	13	60.5 (55.5–65.3)	77.1 (75.8–78.2)	18.0 (16.0–20.1)	95.9 (95.2–96.5)	22
Balkau	5	10.1 (7.4–13.5)	97.4 (96.9–97.8)	24.4 (18.1–31.6)	92.9 (92.1–93.6)	129
Kahn clinic	38	64.0 (59.1–68.6)	74.6 (73.4–75.8)	17.3 (15.4–19.3)	96.1 (95.5–96.7)	20
**Women (n = 2904)**						
CoLaus/PsyCoLaus	13	66.4 (58.3–74.0)	79.8 (78.2–81.3)	15.1 (12.4–18.1)	97.8 (97.1–98.3)	29
Balkau	5	13.4 (8.4–20.0)	97.9 (97.3–98.4)	26.0 (16.6–37.2)	95.4 (94.6–96.2)	145
Kahn clinic	38	62.4 (54.1–70.2)	80.7 (79.1–82.1)	14.9 (12.2–17.9)	97.5 (96.8–98.1)	31
**Men (n = 2373)**						
CoLaus/PsyCoLaus	13	57.0 (50.7–63.2)	73.5 (71.6–75.4)	20.7 (17.7–23.8)	93.4 (92.1–94.5)	16
Balkau	5	8.2 (5.2–12.3)	96.7 (95.8–97.4)	23.1 (14.9–33.1)	89.7 (88.4–90.9)	113
Kahn clinic	38	64.8 (58.7–70.7)	66.8 (64.7–68.8)	19.1 (16.5–21.9)	94.0 (92.7–95.2)	14

PV, predictive value; N, number. Results are expressed as value and (95% confidence interval). Number needed to screen to detect one true incident case of diabetes mellitus was computed as number of participants screened/number of participants who developed diabetes and who scored positive

### Sensitivity analysis

A sensitivity analysis was performed using diabetes based on HbA1c. Of the 4297 participants devoid of diabetes at baseline and who came to the second follow-up, 255 (5.9%) developed diabetes based on HbA1c. The results are summarized in S5 and S6 Tables. Our score showed a higher AUC than the score by Balkau et al., while no differences were found with the score by Kahn et al. (S5 Table), and similar findings were observed for the diagnostic capacity (S6 Table).

Sensitivity analysis after excluding 30 women with personal history of gestational diabetes led to similar findings (S7 and S8 Tables).

### External validation cohorts

The characteristics of the different external validation cohorts are summarized in S9–S11 Tables. The results of our diabetes risk score compared with the other two clinically based diabetes risk scores for each of the three cohorts (European, Tlalpan 2020 and Shahedieh) are summarized in S12 and S13 Tables. Based on the AUC, our score performed better than both scores in the Tlalpan 2020 cohort, better than Kahn et al. on the Shahedieh cohort but performed less well in the European cohort (S12 Table). Our score had a better sensitivity and negative predictive value and a lower specificity and positive predictive value than the score of Balkau et al; the diagnostic capacity was similar to the score by Kahn et al. (S13 Table).

## Discussion

Our score provides an easy way of screening people at risk of developing diabetes; further, and contrary to many other scores [3], it was replicated in other cohorts in Europe and in two developing countries.

### Variables in the model

A previous systematic review identified 29 clinical (i.e. non biological) variables associated with incident diabetes [3]. In this study, we were able to assess the predictive capacity of 19 of them, and six were selected for the final model.

Age was included in the model, as it is in most risk assessment models [8, 15–17]. The risk of diabetes increases with age [18], although in our model no such increase in risk was found. A possible explanation is that other factors such as waist or physical inactivity also increase with age [19, 20], thus cancelling the age-specific increase in diabetes risk.

Gender was also included in the model, as in other scores [7, 17]. Indeed, male sex is associated with a higher risk of diabetes independently of other risk factors [21].

Waist circumference was the obesity measure selected for our score, as it is also used in many other scores [7, 8, 15, 16]. Waist circumference was by far the strongest variable in our score, a finding in agreement with the literature, where abdominal obesity has been found to be the strongest adiposity determinant of diabetes [22, 23]. Importantly, the sole presence of a high waist circumference was enough to consider a subject as at high risk of diabetes, suggesting that waist measurement could already provide important information regarding the risk of diabetes, as it is the case for other populations such as in India [24], or Brazil [25].

Hypertension was included in our score, as it was in many other diabetes risk scores [7, 8, 15–17]. Hypertension is known to be associated with development of diabetes [26–28]. For our score, we defined hypertension as a SBP≥130 mm Hg or DBP ≥85 mm Hg or the presence of antihypertensive drug treatment, but different possible definitions (measured; anamnestic; hypertension medication) have been used [7, 8, 15–17]. We chose to use measured hypertension in our score as this condition is also very prevalent in the general population [29] and is a major risk factor for cardiovascular disease [30]. Hence, assessing risk of diabetes using our score would also help to detect (and manage) hypertension.

Family history of diabetes is generally easy to obtain and was included in our score, as it is in most scores [7, 8, 15, 17]. The presence of a positive family history underlines a genetic component to diabetes but can also reflect the lifestyle or the environmental conditions people were used to during their upbringing [31].

Lack of physical activity was included in the score, as it is also in some other scores. [15, 16]. Physical activity is hard to categorize using a standard procedure; indeed, in this study, it was not possible to obtain a homogeneous definition for physical activity within all cohorts, see S14 Table [32, 33]. Still, irrespective of this limitation, the results were quite similar between cohorts. Hence, our results suggest that even a bold definition of physical activity may suffice to predict the risk of diabetes. This should encourage people to be more active because it underlines that even a small amount of physical activity is better than nothing [34].

### Comparison with other scores

When using CoLaus/PsyCoLaus data, the AUC of our score performed better than the one by Balkau et al. and similarly to Kahn clinic. Our score’s sensitivity was higher than Balkau’s and similar to Kahn’s clinical score. Our specificity was inferior to Balkau’s but similar to Kahn’s clinical score. The positive and negative predictive values were similar to Kahn’s. Although the score by Balkau et al. showed the highest specificity among all cohorts, it led to a considerable underestimation of the prevalence of subjects at risk. For instance, in Iran, the prevalence of subjects at risk according to the score of Balkau et al. was considerably lower than the reported prevalence of diabetes (11.37%) [35].

Overall, our results suggest that our score performs equally well or even better than existing ones. Importantly, the number needed to screen was considerably lower than obtained using the score by Balkau et al., and comparable to the score by Kahn et al. This has important consequences for screening, as is suggests that, for a given number of people screened, a higher number of people who will develop diabetes will be detected.

### Strengths and limitations

The main strength of our study is that the score was replicated on three cohorts from different continents with contrasting diabetes prevalence [36] (S15 Table), a control seldom performed for other scores [3]. Indeed, our score’s AUC in these cohorts is comparable or better than Balkau et al.’s and Kahn et al.’s. Secondly, our score performs similarly to Kahn’s clinical score, but with a smaller number of variables. Importantly, the previous review indicated that many models and scores were not used because they required tests not routinely available or were developed without a specific user in mind [3]. Our score overcomes those two limitations, as it is based on easily accessible data, which could be collected by non-medical professionals. Hence, it could be implemented worldwide with little effort. A third strength is that obesity prevalence in women was different in each replication cohort, which reinforces our score. In men, however, average waist circumference was very similar among the cohorts. Fourthly, although part of the type 2 diabetes epidemic is due to environmental factors, our score is important on a personal level. Indeed, if one calculates one’s diabetes score and it is elevated, every individual can decide to act upon the three modifiable variables (hypertension, waist circumference and physical activity). Fifthly, the scoring system could be adapted to the characteristics of the populations, by changing the threshold and/or the weights of its parameters, as it has been done for cardiovascular risk scores [37]. Sixthly, the American Diabetes Assocation (ADA) recommends testing all people, beginning at age 45 years.[38] The CoLaus/PsyColaus sample included adults aged 35 to 75, which is within the recommended age frame. Finally, our score includes 4 of the 9 criteria issued by the ADA recommendations for testing diabetes or prediabetes in asymptomatic adults: overweight, hypertension, family history, physical inactivity.[38] This shows the importance of these variables in the development of diabetes.

This study also has some limitations worth acknowledging. Firstly, participants excluded from the analysis presented higher prevalence of several components of the diabetes risk score. Hence, it is likely that the impact of some components might have been different, had those participants been included in the analysis. Still, external validation of the analysis in other cohorts led to similar findings. Secondly, the number of incident diabetes cases was relatively low in the Shahedieh cohort, a finding likely due to a short follow-up time (1 year) and leading to a lower statistical power. Hence, it would be of interest to replicate the analysis in the forthcoming years with a larger number of incident diabetes cases. Thirdly, we were unable to validate our score on a South-Eastern Asian country, where waist circumference is considered as a major determinant of diabetes [39]. Still, given the importance of waist circumference in our score, we hypothesize that it would perform relatively well in those countries, although an external validation study in a South-Eastern Asian country is necessary to test this hypothesis, especially in the genetically lean Asian men. Fourthly, physical activity was assessed differently in each cohort (S14 Table). Assessing physical activity in a standardized manner is a difficult task, and there is even lack of standardization when physical activity is measured using accelerometry [40]. Hence, we chose a very pragmatic solution, where each cohort would define physical activity according to its own standards. We acknowledge that this procedure increases variability between cohorts, but on the other hand it allows the use of our score by many other cohorts. Fifthly, our score uses a fixed weight for all risk factors independently of the country considered, while it has been suggested that the importance of conventional risk factors for predicting diabetes varies between countries [41]. Indeed, levels of diabetes have been shown to vary according to socio-economic status [42] and ethnicity [43]. However, in our study, neither ethnicity nor education came out as significant predictors for type 2 diabetes. Further, waist circumference has been shown to be the best anthropometric predictor across all racial and ethnic groups [44]. Moreover, due to its simple scoring system, our score allows the addition of other risk factors if the latter are considered as important in a given setting. Alternatively, it might be necessary to recalibrate our score according to country, as it has been suggested for cardiovascular risk prediction [37]. Finally, as other scores, ours did not allow the identification of all incident cases of diabetes. Still, it can be used with minimal clinical data, and can thus be applied in settings with limited health resources where no screening for diabetes risk is available.

### Conclusion

Our clinically-based score shows comparable or even better results to other clinical scores and can be used on different populations with contrasting diabetes prevalence.

## Supporting information

S1 Table Characteristics of the diabetes risk scores. (DOCX)

S2 Table Baseline characteristics of included and excluded participants, CoLaus/PsyCoLaus study, Lausanne, Switzerland. (DOCX)

S3 Table Results of the logistic regression using incident diabetes as dependent variable, both genders, CoLaus/PsyCoLaus study. (DOCX)

S4 Table Goodness of fit and information criteria for each diabetes risk score, overall and stratified by gender, CoLaus/PsyCoLaus study, Lausanne, Switzerland. (DOCX)

S5 Table Areas under the ROC and incidence of diabetes as defined by glycated haemoglobin, per quintile of diabetes risk estimation score, overall and stratified by gender, CoLaus/PsyCoLaus study, Lausanne, Switzerland, 2003–2017. (DOCX)

S6 Table Diagnostic capacity of the different scores, overall and stratified by gender, using diabetes as defined by glycated haemoglobin, CoLaus/PsyCoLaus study, Lausanne, Switzerland, 2003–2017. (DOCX)

S7 Table Areas under the receiver operating characteristic (ROC) curves and incidence of diabetes per quintile of diabetes risk estimation score, women without history of gestational diabetes (n = 2874), CoLaus/PsyCoLaus study, Lausanne, Switzerland, 2003–2017. (DOCX)

S8 Table Diagnostic capacity of the different scores, women without history of gestational diabetes (n = 2874), CoLaus/PsyCoLaus study, Lausanne, Switzerland, 2003–2017. (DOCX)

S9 Table Characteristics of the four countries (France, Germany, Netherlands and UK) contributing data from the EPIC-Europe cohort study. (DOCX)

S10 Table Characteristics of the Tlalpan 2020 cohort, Mexico City, Mexico. (DOCX)

S11 Table Characteristics of the Shahedieh cohort, Iran. (DOCX)

S12 Table Performance of the new score and of two other clinically based scores, in original cohort (CoLaus/PsyCoLaus) and in the replication cohorts. (DOCX)

S13 Table Diagnostic capacity of the new score and of two other clinically based scores, in original cohort (CoLaus/PsyCoLaus) and in the replication cohorts. (DOCX)

S14 Table Definitions of physical activity in the original and in the replication cohorts. (DOCX)

S15 Table Diabetes prevalence in percentage of adults in 2014 classified by country and gender. (DOCX)
