# Supplementary material for: Development and multi-cohort validation of a clinical score for predicting type 2 diabetes mellitus
Source: PLoS One. 2019 Oct 9;14(10):e0218933. doi: 10.1371/journal.pone.0218933 (PMC6785081; doi:10.1371/journal.pone.0218933)
Supplement: S1 Table — (DOCX) [file pone.0218933.s001.docx]

Supplemental information

**S1 Table. Characteristics of the diabetes risk scores**

|  | **CoLaus/PsyCoLaus score** | **Balkau et al.^7^** | **Kahn et al (C)^8^** |
| --- | --- | --- | --- |
| Country |  | France | USA |
| Population number |  | 3817 | 12’729 |
| Follow-up (years) |  | 9 | 9 |
| Age | X |  | X |
| Sex | X | X |  |
| BMI, Weight |  |  | X |
| Waist | X | X | X |
| Height |  |  | X |
| Hypertension* | HM/MH | HM/MH | HM/MH |
| Resting pulse |  |  | X |
| Family history of diabetes | X | X | X |
| History of hyperglycemia |  |  |  |
| Steroids |  |  |  |
| Physical activity | X |  |  |
| Smoking |  | X | X |
| Alcohol |  |  |  |
| Fruit & vegetable consumption |  |  |  |
| Glucose level |  |  |  |
| Triglycerides |  |  |  |
| High density lipoproteins |  |  |  |
| Uric acid |  |  |  |
| Number of variables | 6 | 5 | 8 |

*****AH: anamnestic hypertension, HM: hypertension medication; MH measured hypertension; NA, not available
