# Supplementary material for: Development and multi-cohort validation of a clinical score for predicting type 2 diabetes mellitus
Source: PLoS One. 2019 Oct 9;14(10):e0218933. doi: 10.1371/journal.pone.0218933 (PMC6785081; doi:10.1371/journal.pone.0218933)
Supplement: S2 Table — (DOCX) [file pone.0218933.s002.docx]

Supplemental information

**S2 Table. Baseline characteristics of included and excluded participants, CoLaus/PsyCoLaus study, Lausanne, Switzerland.**

|  | **Included** | **Excluded** | **P-value** |
| --- | --- | --- | --- |
| Sample size | 5277 | 1456 |  |
| Woman | 2904 (55.0) | 640 (44.0) | <0.001 |
| Age (years) | 51.8 ± 10.5 | 55.4 ± 11.2 | <0.001 |
| Anthropometry |  |  |  |
| Height (cm) | 168 ± 9 | 168 ± 9 | 0.399 |
| Weight (kg) | 72.2 ± 14.3 | 77.6 ± 17.1 | <0.001 |
| Body mass index (kg/cm^2^) | 25.4 ± 4.2 | 27.4 ± 5.3 | <0.001 |
| Waist circumference (cm) | 87.7 ± 12.5 | 94.1 ± 14.8 | <0.001 |
| Waist to height ratio | 52.1 ± 7.0 | 56.0 ± 8.5 | <0.001 |
| Waist to hip ratio | 0.87 ± 0.08 | 0.91 ± 0.09 | <0.001 |
| Educational level |  |  | <0.001 |
| University | 1121 (21.2) | 199 (13.8) |  |
| High school | 1341 (25.4) | 284 (19.7) |  |
| Apprenticeship | 1867 (35.4) | 510 (35.4) |  |
| Mandatory education | 948 (18.0) | 449 (31.1) |  |
| Smoking status |  |  | 0.012 |
| Never | 2182 (41.4) | 550 (37.9) |  |
| Former | 1715 (32.5) | 468 (32.3) |  |
| Current | 1380 (26.2) | 432 (29.8) |  |
| Blood pressure ≥ 130/85 mm Hg | 2253 (42.7) | 825 (57.0) | <0.001 |
| Hypertension † | 2457 (46.6) | 921 (63.6) | <0.001 |
| High resting heart rate ‡ | 2236 (42.4) | 731 (50.6) | <0.001 |
| Lipid lowering drugs | 508 (9.6) | 277 (19.0) | <0.001 |
| Alcohol ≥35 drinks/week | 83 (1.6) | 47 (3.2) | <0.001 |
| Caffeinated drinks (units/day) |  |  | 0.001 |
| None | 331 (6.3) | 119 (8.2) |  |
| 1.3 | 3433 (65.1) | 951 (65.6) |  |
| 4-6 | 1276 (24.2) | 297 (20.5) |  |
| >6 | 237 (4.5) | 82 (5.7) |  |
| Leisure-time PA ≥2/week | 2942 (55.8) | 653 (45.0) | <0.001 |
| Personal history of CVD | 266 (5.0) | 141 (9.7) | <0.001 |
| Parental history of diabetes |  |  | <0.001 |
| No | 4333 (82.1) | 1130 (77.6) |  |
| Mother only | 483 (9.2) | 184 (12.6) |  |
| Father only | 424 (8.0) | 125 (8.6) |  |
| Both | 37 (0.7) | 17 (1.2) |  |
| Family history of diabetes (type 1+2) | 1151 (21.8) | 421 (28.9) | <0.001 |

CVD, cardiovascular disease; PA, physical activity. † defined by SBP≥130 mm Hg or DBP ≥85 mm Hg or presence of antihypertensive drug treatment; ‡, defined by ≥68 beats per minute in men and ≥70 beats per minute in women.

Results are expressed as mean ± standard deviation or as number of participants (%). Between-group comparisons performed using student’s t-test for continuous variables and chi-square for categorical variables.
