# Supplementary material for: Development and multi-cohort validation of a clinical score for predicting type 2 diabetes mellitus
Source: PLoS One. 2019 Oct 9;14(10):e0218933. doi: 10.1371/journal.pone.0218933 (PMC6785081; doi:10.1371/journal.pone.0218933)
Supplement: S3 Table — (DOCX) [file pone.0218933.s003.docx]

Supplemental information

**S3 Table. Results of the logistic regression using incident diabetes as dependent variable, both genders, CoLaus/PsyCoLaus study.**

|  | **Odds ratio (95% confidence** | **P-value** |
| --- | --- | --- |
| Male gender | 2.18 (1.73 - 2.73) | <0.001 |
| Age group (years) |  |  |
| [45-54] | 1.87 (1.34 - 2.60) | <0.001 |
| [55-64] | 2.18 (1.56 - 3.06) | <0.001 |
| [65-75] | 1.83 (1.24 - 2.71) | 0.002 |
| Waist (cm) |  |  |
| 70-79 W, 80-89 M | 2.50 (0.88 - 7.04) | 0.084 |
| 80-89 W, 90-99 M | 6.08 (2.22 - 16.7) | <0.001 |
| 90-99 W, 100-109 M | 12.1 (4.40 - 33.1) | <0.001 |
| 100-109 W, 110-120 M | 18.1 (6.44 - 50.7) | <0.001 |
| 110+ W, 120+ M | 31.8 (10.7 - 94.4) | <0.001 |
| Family history of diabetes § | 1.90 (1.50 - 2.39) | <0.001 |
| Physical inactivity ǂ | 1.32 (1.06 - 1.64) | 0.013 |
| Hypertension † | 1.61 (1.25 - 2.07) | <0.001 |

§: father, mother or siblings; ǂ: less than twice 20 minutes leisure physical activity per week; †: systolic blood pressure ≥ 130 mm Hg and/or diastolic blood pressure ≥ 85 mmHg and/or antihypertensive drug treatment. Results are expressed as odds ratio and (95% confidence interval).
