# Supplementary material for: Development and multi-cohort validation of a clinical score for predicting type 2 diabetes mellitus
Source: PLoS One. 2019 Oct 9;14(10):e0218933. doi: 10.1371/journal.pone.0218933 (PMC6785081; doi:10.1371/journal.pone.0218933)
Supplement: S4 Table — (DOCX) [file pone.0218933.s004.docx]

Supplemental information

**S4 Table. Goodness of fit and information criteria for each diabetes risk score, overall and stratified by gender, CoLaus/PsyCoLaus study, Lausanne, Switzerland.**

|  | **Goodness of fit ǂ** | **Akaike IC** | **Bayesian IC** |
| --- | --- | --- | --- |
| **All participants** |  |  |  |
| CoLaus | <0.001 | 2559.00 | 2572.14 |
| Balkau | 0.009 | 2552.36 | 2565.50 |
| Kahn clinic | 0.032 | 2491.39 | 2504.53 |
| **Women (n=2904)** |  |  |  |
| CoLaus | 0.022 | 1027.01 | 1038.96 |
| Balkau | 0.816 | 1012.74 | 1024.69 |
| Kahn clinic | 0.046 | 992.69 | 1004.64 |
| **Men (n=2373)** |  |  |  |
| CoLaus | <0.001 | 1504.31 | 1515.85 |
| Balkau | 0.013 | 1506.33 | 1517.87 |
| Kahn clinic | 0.047 | 1477.01 | 1488.55 |

ǂ p-value of Hosmer-Lemeshow test using 10 groups
