# Supplementary material for: Development and multi-cohort validation of a clinical score for predicting type 2 diabetes mellitus
Source: PLoS One. 2019 Oct 9;14(10):e0218933. doi: 10.1371/journal.pone.0218933 (PMC6785081; doi:10.1371/journal.pone.0218933)
Supplement: S5 Table — (DOCX) [file pone.0218933.s005.docx]

Supplemental information

**S5 Table. Areas under the ROC and incidence of diabetes as defined by glycated haemoglobin, per quintile of diabetes risk estimation score, overall and stratified by gender, CoLaus/PsyCoLaus study, Lausanne, Switzerland, 2003-2017.**

|  | **AUC (95% CI)** | **P-value §** | **First** | **Second** | **Third** | **Fourth** | **Fifth** |
| --- | --- | --- | --- | --- | --- | --- | --- |
| **All participants (4297)** |  |  |  |  |  |  |  |
| CoLaus/PsyCoLaus | 0.802 (0.776 - 0.827) |  | 0.6 | 1.8 | 4.3 | 7.8 | 18.2 |
| Balkau | 0.775 (0.750 - 0.801) | 0.001 | 0.5 | - | 3.4 | 6.9 | 17.7 |
| Kahn clinic | 0.805 (0.780 - 0.830) | 0.596 | 0.4 | 1.2 | 4.2 | 7.0 | 17.5 |
| **Women (n=2413)** |  |  |  |  |  |  |  |
| CoLaus/PsyCoLaus | 0.834 (0.799 - 0.869) |  | 0.3 | 1.8 | 3.8 | 5.3 | 17.6 |
| Balkau | 0.817 (0.781 - 0.852) | 0.070 | 0.2 | - | 2.3 | 5.4 | 15.8 |
| Kahn clinic | 0.840 (0.808 - 0.872) | 0.544 | 0.0 | 0.6 | 4.5 | 4.7 | 17.2 |
| **Men (n=1884)** |  |  |  |  |  |  |  |
| CoLaus/PsyCoLaus | 0.757 (0.718 - 0.795) |  | 1.5 | 1.7 | 4.6 | 9.9 | 18.8 |
| Balkau | 0.728 (0.691 - 0.765) | 0.026 | 1.1 | - | 4.8 | 8.3 | 19.4 |
| Kahn clinic | 0.762 (0.724 - 0.799) | 0.580 | 1.3 | 2.2 | 3.8 | 9.5 | 17.7 |

AUC, area under the ROC. Results are expressed as value and (95% confidence interval) for AUC and as percentage for the incidence of diabetes. Comparison of AUC performed using the **roccomp** procedure of Stata.
