# Supplementary material for: Development and multi-cohort validation of a clinical score for predicting type 2 diabetes mellitus
Source: PLoS One. 2019 Oct 9;14(10):e0218933. doi: 10.1371/journal.pone.0218933 (PMC6785081; doi:10.1371/journal.pone.0218933)
Supplement: S6 Table — (DOCX) [file pone.0218933.s006.docx]

Supplemental information

**S6 Table. Diagnostic capacity of the different scores, overall and stratified by gender, using diabetes as defined by glycated haemoglobin, CoLaus/PsyCoLaus study, Lausanne, Switzerland, 2003-2017.**

|  | **Threshold** | **Sensitivity** | **Specificity** | **Positive predictive value** | **Negative predictive value** | **Number needed to screen** |
| --- | --- | --- | --- | --- | --- | --- |
| **All participants** |  |  |  |  |  |  |
| CoLaus/PsyCoLaus | 13 | 65.5 (59.3 - 71.3) | 77.6 (76.3 - 78.9) | 15.6 (13.5 - 17.9) | 97.3 (96.6 - 97.8) | 26 |
| Balkau | 5 | 10.6 (7.1 - 15.0) | 97.6 (97.0 - 98.0) | 21.4 (14.6 - 29.6) | 94.5 (93.8 - 95.2) | 159 |
| Kahn clinic | 38 | 68.6 (62.5 - 74.3) | 76.0 (74.6 - 77.3) | 15.3 (13.2 - 17.5) | 97.5 (96.8 - 98.0) | 25 |
| **Women (n=2904)** |  |  |  |  |  |  |
| CoLaus/PsyCoLaus | 13 | 72.1 (62.5 - 80.5) | 80.0 (78.3 - 81.6) | 14.0 (11.2 - 17.2) | 98.5 (97.8 - 99.0) | 32 |
| Balkau | 5 | 15.4 (9.1 - 23.8) | 98.1 (97.4 - 98.6) | 26.2 (15.8 - 39.1) | 96.3 (95.4 - 97.0) | 151 |
| Kahn clinic | 38 | 68.3 (58.4 - 77.1) | 81.0 (79.4 - 82.6) | 13.9 (11.1 - 17.3) | 98.3 (97.6 - 98.8) | 34 |
| **Men (n=2373)** |  |  |  |  |  |  |
| CoLaus/PsyCoLaus | 13 | 60.9 (52.7 - 68.8) | 74.4 (72.3 - 76.5) | 17.2 (14.1 - 20.7) | 95.6 (94.4 - 96.7) | 20 |
| Balkau | 5 | 7.3 (3.7 - 12.7) | 96.9 (96.0 - 97.7) | 16.9 (8.8 - 28.3) | 92.3 (91.0 - 93.5) | 171 |
| Kahn clinic | 38 | 68.9 (60.8 - 76.2) | 69.2 (67.0 - 71.4) | 16.3 (13.5 - 19.4) | 96.2 (95.0 - 97.2) | 18 |

Results are expressed as value (95% confidence interval), except for the number needed to screen. Number needed to screen to detect one true incident case of diabetes mellitus: total number of participants screened/number of participants who developed T2DM and who scored positive.
