# Supplementary material for: Development and multi-cohort validation of a clinical score for predicting type 2 diabetes mellitus
Source: PLoS One. 2019 Oct 9;14(10):e0218933. doi: 10.1371/journal.pone.0218933 (PMC6785081; doi:10.1371/journal.pone.0218933)
Supplement: S7 Table — (DOCX) [file pone.0218933.s007.docx]

Supplemental information

**S7 Table. Areas under the receiver operating characteristic (ROC) curves and incidence of diabetes per quintile of diabetes risk estimation score, women without history of gestational diabetes (n=2874), CoLaus/PsyCoLaus study, Lausanne, Switzerland, 2003-2017**

|  | **AUC (95% CI)** | **P-value §** | **First** | **Second** | **Third** | **Fourth** | **Fifth** |
| --- | --- | --- | --- | --- | --- | --- | --- |
| CoLaus/PsyCoLaus | 0.804 (0.769 - 0.838) |  | 0.8 | 3.3 | 4.7 | 9.1 | 21.4 |
| Balkau | 0.786 (0.751 - 0.820) | 0.029 | 0.8 | 2.8 | 6.3 | NA | 16.6 |
| Kahn clinic | 0.805 (0.772 - 0.838) | 0.903 | 0.4 | 1.6 | 2.5 | 6.3 | 15.3 |

Results are expressed as area under the curve and (95% confidence interval), and as percentage of participants developing diabetes during the 10.9 year follow-up. Diabetes was defined as fasting glucose level ≥7 mmol/L and/or presence of an oral antidiabetic or insulin treatment.
