# Supplementary material for: Development and multi-cohort validation of a clinical score for predicting type 2 diabetes mellitus
Source: PLoS One. 2019 Oct 9;14(10):e0218933. doi: 10.1371/journal.pone.0218933 (PMC6785081; doi:10.1371/journal.pone.0218933)
Supplement: S8 Table — (DOCX) [file pone.0218933.s008.docx]

Supplemental information

**S8 Table. Diagnostic capacity of the different scores, women without history of gestational diabetes (n=2874), CoLaus/PsyCoLaus study, Lausanne, Switzerland, 2003-2017.**

|  | **Threshold** | **Sensitivity** | **Specificity** | **Positive PV** | **Negative PV** | **N needed to screen** |
| --- | --- | --- | --- | --- | --- | --- |
| CoLaus/PsyCoLaus | 13 | 65.8 (57.5 - 73.4) | 79.7 (78.2 - 81.2) | 14.8 (12.1 - 17.8) | 97.8 (97.0 - 98.3) | 30 |
| Balkau | 5 | 13.7 (8.6 - 20.4) | 97.9 (97.3 - 98.4) | 26.0 (16.6 - 37.2) | 95.5 (94.7 - 96.2) | 144 |
| Kahn clinic | 38 | 62.3 (53.9 - 70.2) | 80.5 (79.0 - 82.0) | 14.6 (11.9 - 17.7) | 97.6 (96.8 - 98.2) | 32 |

PV, predictive value; N, number. Results are expressed as value and (95% confidence interval). Number needed to screen to detect one true incident case of diabetes mellitus was computed as number of participants screened/number of participants who developed diabetes and who scored positive
