# Supplementary material for: Development and multi-cohort validation of a clinical score for predicting type 2 diabetes mellitus
Source: PLoS One. 2019 Oct 9;14(10):e0218933. doi: 10.1371/journal.pone.0218933 (PMC6785081; doi:10.1371/journal.pone.0218933)
Supplement: S9 Table — (DOCX) [file pone.0218933.s009.docx]

Supplemental information

**S9 Table. Characteristics of the four countries (France, Germany, Netherlands and UK) contributing data from the EPIC-Europe cohort study**

|  | **Men** | **Women** | **All** |
| --- | --- | --- | --- |
| Sample size | 13,469 (21.9) | 48,126 (78.1) | 61,595 |
| Age (years) | 57.8 ± 8.2 | 56.2 ± 7.0 | 56.6 ± 7.3 |
| Anthropometry |  |  |  |
| Height (cm) | 175 ± 7 | 162 ± 6 | 165 ± 8 |
| Weight (kg) | 80.3 ± 11.8 | 65.4 ± 11.6 | 68.7 ± 13.2 |
| Body mass index (kg/cm^2^) | 26.3 ± 3.4 | 24.8 ± 4.1 | 25.1 ± 4.0 |
| Waist circumference (cm) | 94.0 ± 9.9 | 79.6 ± 10.3 | 82.8 ± 11.8 |
| Smoking status |  |  |  |
| Never | 3963 (29.4) | 26763 (55.6) | 30726 (49.9) |
| Former | 6397 (47.5) | 13927 (28.9) | 20324 (33.0) |
| Current | 3109 (23.1) | 7436 (15.5) | 10545 (17.1) |
| Hemodynamic |  |  |  |
| Heart rate (bpm) | 70 ± 12 | 72 ± 11 | 72 ± 11 |
| Hypertension † | 8629 (64.1) | 24846 (51.6) | 33475 (54.3) |
| Hypertension ǂ | 5805 (43.1) | 16533 (34.4) | 22338 (36.3) |
| Family history of diabetes |  |  |  |
| All family members | 1998 (14.8) | 9041 (18.8) | 11039 (17.9) |
| Father | 685 (5.1) | 3274 (6.8) | 3959 (6.4) |
| Mother | 1107 (8.2) | 5281 (11.0) | 6388 (10.4) |
| Physical inactivity | 5917 (43.9) | 22978 (47.7) | 28895 (46.9) |

BP, blood pressure; CVD, cardiovascular disease. † defined by SBP≥130 mm Hg or DBP ≥85 mm Hg or presence of antihypertensive drug treatment. ǂ defined by SBP≥140 mm Hg or DBP ≥90 mm Hg or presence of antihypertensive drug treatment. Results are expressed as mean ± standard deviation or as number of participants (%).
