# Supplementary material for: Development and multi-cohort validation of a clinical score for predicting type 2 diabetes mellitus
Source: PLoS One. 2019 Oct 9;14(10):e0218933. doi: 10.1371/journal.pone.0218933 (PMC6785081; doi:10.1371/journal.pone.0218933)
Supplement: S10 Table — (DOCX) [file pone.0218933.s010.docx]

Supplemental information

**S10 Table. Characteristics of the Tlalpan 2020 cohort, Mexico City, Mexico.**

|  | **Men** | **Women** | **All** |
| --- | --- | --- | --- |
| Sample size | 405 (33.6) | 798 (66.4) | 1203 |
| Age (years) | 43.2 ± 4.6 | 43.3 ± 4.6 | 43.3 ± 4.6 |
| Anthropometry |  |  |  |
| Height (m) | 170.1 ± 6.3 | 156.8 ± 6.2 | 161.3 ± 8.9 |
| Weight (kg) | 81.8 ± 13.7 | 67.9 ± 12.8 | 72.6 ± 14.7 |
| Body mass index (kg/cm^2^) | 28.2 ± 4.2 | 27.6 ± 4.8 | 27.8 ± 4.6 |
| Waist circumference (cm) | 97.7 ± 11.0 | 89.9 ± 11.6 | 92.5 ± 12.0 |
| Hemodynamic |  |  |  |
| Heart rate (bpm) | 64 ± 8 | 65 ± 8 | 65 ± 8 |
| Hypertension † | 79 (19.5) | 50 (6.3) | 129 (10.7) |
| Hypertension ǂ | 26 (6.4) | 15 (1.9) | 41 (3.4) |
| Smoking status |  |  |  |
| Never | 210 (51.9) | 420 (52.6) | 630 (52.4) |
| Former | 107 (26.4) | 189 (23.7) | 296 (24.6) |
| Current | 88 (21.7) | 189 (23.7) | 277 (23.0) |
| Family history of diabetes |  |  |  |
| All family members | 155 (38.3) | 377 (47.2) | 532 (44.2) |
| Father | 98 (24.2) | 221 (27.7) | 319 (26.5) |
| Mother | 83 (20.5) | 225 (28.2) | 308 (25.6) |
| Physical inactivity | 359 (88.6) | 744 (93.2) | 1,103 (91.7) |

BP, blood pressure; CVD, cardiovascular disease. † defined by SBP≥130 mm Hg or DBP ≥85 mm Hg or presence of antihypertensive drug treatment. ǂ defined by SBP≥140 mm Hg or DBP ≥90 mm Hg or presence of antihypertensive drug treatment. Results are expressed as mean ± standard deviation or as number of participants (%).
