# Supplementary material for: Development and multi-cohort validation of a clinical score for predicting type 2 diabetes mellitus
Source: PLoS One. 2019 Oct 9;14(10):e0218933. doi: 10.1371/journal.pone.0218933 (PMC6785081; doi:10.1371/journal.pone.0218933)
Supplement: S11 Table — (DOCX) [file pone.0218933.s011.docx]

Supplemental information

**S11 Table. Characteristics of the Shahedieh cohort, Iran.**

|  | **Men** | **Women** | **All** |
| --- | --- | --- | --- |
| Sample size | 5030 (50.4) | 4945 (49.6) | 9975 (100) |
| Age (years) | 50.4±9.7 | 50.8±9.6 | 50.6±9.6 |
| Anthropometry |  |  |  |
| Height (cm) | 170.5 ± 6.7 | 156.1 ± 6.0 | 163.4 ± 9.6 |
| Weight (kg) | 79.1 ± 13.9 | 72.4 ± 13.1 | 75.8 ± 13.9 |
| Body mass index (kg/cm^2^) | 27.2 ± 4.4 | 29.7 ± 5.1 | 28.4 ± 4.9 |
| Waist circumference (cm) | 94.7 ± 11.4 | 97.5 ± 12 | 96.1 ± 11.7 |
| Hemodynamic |  |  |  |
| Heart rate (bpm) | 73 ± 9 | 74 ± 9 | 74 ± 9 |
| Hypertension † | 728 (14.7) | 786 (16.1) | 1514 (15.4) |
| Hypertension ǂ | 367 (7.4) | 392 (8.0) | 759 (7.7) |
| Smoking status |  |  |  |
| Never | 3952 (78.6) | 4934 (99.8) | 8886 (88.1) |
| Former | 304 (6.0) | 5 (0.1) | 309 (3.1) |
| Current | 774 (15.4) | 6 (0.12) | 780 (7.8) |
| Parental history of diabetes |  |  |  |
| All family members | 2550 (51.2) | 2489 (50.9) | 5039 (51.0) |
| Father | 1021 (20.5) | 1014 (20.7) | 2035 (20.6) |
| Mother | 665 (13.4) | 670 (13.7) | 1335 (13.5) |
| Physical inactivity* | 4484 (90.0) | 4383 (89.6) | 8867 (89.8) |

BP, blood pressure; CVD, cardiovascular disease. † defined by SBP≥130 mm Hg or DBP ≥85 mm Hg or presence of antihypertensive drug treatment. ǂ defined by SBP≥140 mm Hg or DBP ≥90 mm Hg or presence of antihypertensive drug treatment. Results are expressed as mean ± standard deviation or as number of participants (%). *Less than 10% of day time moderate to high physical activity.
