# Supplementary material for: Development and multi-cohort validation of a clinical score for predicting type 2 diabetes mellitus
Source: PLoS One. 2019 Oct 9;14(10):e0218933. doi: 10.1371/journal.pone.0218933 (PMC6785081; doi:10.1371/journal.pone.0218933)
Supplement: S12 Table — (DOCX) [file pone.0218933.s012.docx]

Supplemental information

**S12 Table. Performance of the new score and of two other clinically based scores, in original cohort (CoLaus/PsyCoLaus) and in the replication cohorts.**

|  | **AUC (95% CI)** | **p-value §** | **Goodness of fit †** | **AIC** | **BIC** |
| --- | --- | --- | --- | --- | --- |
| CoLaus/PsyCoLaus |  |  |  |  |  |
| CoLaus/PsyCoLaus | 0.772 (0.750 - 0.794) |  | <0.001 | 2559.0 | 2572.1 |
| Balkau | 0.748 (0.726 - 0.770) | <0.001 | 0.009 | 2552.4 | 2565.5 |
| Kahn clinic | 0.774 (0.753 - 0.796) | 0.668 | 0.032 | 2491.4 | 2504.5 |
| European |  |  |  |  |  |
| CoLaus/PsyCoLaus | 0.788 (0.779 - 0.798) |  | <0.001 | 17419.3 | 17437.4 |
| Balkau | 0.793 (0.784 - 0.801) | 0.091 | <0.001 | 16855.6 | 16873.6 |
| Kahn clinic | 0.816 (0.807 - 0.825) | <0.001 | 0.036 | 16325.9 | 16343.9 |
| Tlalpan 2020 |  |  |  |  |  |
| CoLaus/PsyCoLaus | 0.791 (0.684 - 0.898) |  | 0.716 | 214.2 | 224.4 |
| Balkau | 0.672 (0.561 - 0.782) | <0.001 | 0.221 | 236.3 | 246.5 |
| Kahn clinic | 0.778 (0.660 - 0.895) | 0.575 | 0.025 | 216.5 | 226.7 |
| Shahedieh |  |  |  |  |  |
| CoLaus/PsyCoLaus | 0.542 (0.459-0.626) |  | 0.814 | 588.2 | 602.6 |
| Balkau | 0.564 (0.481-0.646) | 0.476 | 0.358 | 587.5 | 601.9 |
| Kahn clinic | 0.513 (0.431-0.595) | 0.300 | 0.702 | 589.7 | 604.1 |

§ comparing the model to the new one; † p-value for the Hosmer-Lemeshow test using 10 categories. AIC, Akaike’s information criterion; BIC, Bayesian information criterion
