# Supplementary material for: Development and multi-cohort validation of a clinical score for predicting type 2 diabetes mellitus
Source: PLoS One. 2019 Oct 9;14(10):e0218933. doi: 10.1371/journal.pone.0218933 (PMC6785081; doi:10.1371/journal.pone.0218933)
Supplement: S13 Table — (DOCX) [file pone.0218933.s013.docx]

Supplemental information

**S13 Table. Diagnostic capacity of the new score and of two other clinically based scores, in original cohort (CoLaus/PsyCoLaus) and in the replication cohorts.**

|  | **Threshold** | **Sensitivity** | **Specificity** | **Positive predictive value** | **Negative predictive value** |
| --- | --- | --- | --- | --- | --- |
| CoLaus/PsyCoLaus |  |  |  |  |  |
| CoLaus/PsyCoLaus | 13 | 60.5 (55.5 - 65.3) | 77.1 (75.8 – 78.2) | 18.0 (16.0 – 20.1) | 95.9 (95.2 – 96.5) |
| Balkau | 5 | 10.1 (7.4 – 13.5) | 97.4 (96.9 – 97.8) | 24.4 (18.1 – 31.6) | 92.9 (92.1 – 93.6) |
| Kahn clinic | 38 | 64.0 (59.1 – 68.6) | 74.6 (73.4 – 75.8) | 17.3 (15.4 – 19.3) | 96.1 (95.5 – 96.7) |
| European |  |  |  |  |  |
| CoLaus/PsyCoLaus | 13 | 59.3 (57.2 - 61.3) | 82.7 (82.4 - 83.0) | 11.7 (11.1 - 12.3) | 98.1 (98.0 - 98.3) |
| Balkau | 5 | 13.2 (11.9 - 14.7) | 98.0 (97.9 - 98.1) | 20.2 (18.2 - 22.3) | 96.7 (96.6 - 96.8) |
| Kahn clinic | 38 | 69.7 (67.7 - 71.5) | 79.0 (78.6 - 79.3) | 11.3 (10.8 - 11.9) | 98.5 (98.4 - 98.6) |
| Tlalpan 2020 |  |  |  |  |  |
| CoLaus/PsyCoLaus | 13 | 84.0 (63.9 - 95.5) | 53.1 (50.2 - 56.0) | 3.7 (2.3 - 5.6) | 99.4 (98.4 - 99.8) |
| Balkau | 5 | 8.0 (1.0 - 26.0) | 99.4 (98.8 - 99.8) | 22.2 (2.8 - 60.0) | 98.1 (97.1 - 98.8) |
| Kahn clinic | 38 | 76.0 (54.9 - 90.6) | 61.3 (58.4 - 64.1) | 4.0 (2.4 - 6.2) | 99.2 (98.2 - 99.7) |
| Shahedieh |  |  |  |  |  |
| CoLaus/PsyCoLaus | 13 | 63.0 (47.5-76.8) | 47.0 (46.0-48.0) | 0.6 (0.4-0.8) | 99.6 (99.4-99.8) |
| Balkau | 5 | 0.0 (0.0-7.7) | 98.3 (98.0-98.6) | 0.0 (0.0-2.2) | 99.5 (99.4-99.7) |
| Kahn clinic | 38 | 50.0 (34.9-65.1) | 52.7 (51.7-53.7) | 0.5 (0.3-0.7) | 99.6 (99.3-99.7) |

Results are expressed as value and (95% confidence interval).
