# Supplementary material for: Development and multi-cohort validation of a clinical score for predicting type 2 diabetes mellitus
Source: PLoS One. 2019 Oct 9;14(10):e0218933. doi: 10.1371/journal.pone.0218933 (PMC6785081; doi:10.1371/journal.pone.0218933)
Supplement: S14 Table — (DOCX) [file pone.0218933.s014.docx]

Supplemental information

**S14 Table. Definitions of physical activity in the original and in the replication cohorts.**

| **Cohort** | **Physical inactivity** | **Physical Activity** |
| --- | --- | --- |
| CoLaus/PsyCoLaus | Less than twice 20 minutes leisure physical activity/week | ≥twice 20 minutes leisure physical activity/week |
| European | Inactive and moderately inactive in the self-reported Cambridge physical activity index^32^ | Active and moderately active in the self-reported Cambridge physical activity index^32^ |
| Tlalpan 2020 | Low IPAQ score^33^ | Moderate or high IPAQ score^33^ |
| Shahedieh | Moderate and severe physical activity < 10% of the day | Moderate and severe physical activity ≥10% of the day |
