# Supplementary material for: Development and multi-cohort validation of a clinical score for predicting type 2 diabetes mellitus
Source: PLoS One. 2019 Oct 9;14(10):e0218933. doi: 10.1371/journal.pone.0218933 (PMC6785081; doi:10.1371/journal.pone.0218933)
Supplement: S15 Table — (DOCX) [file pone.0218933.s015.docx]

Supplemental information

**S15 Table. Diabetes prevalence in percentage of adults in 2014 classified by country and gender**^36^

|  | **Men** | **Women** |
| --- | --- | --- |
| United Kingdom | 6.6 (4.1-9.7) | 4.9 (3.1-7.4) |
| France | 7.5 (4.1-12.2) | 4.4 (2.3-7.5) |
| Germany | 6.0 (3.3-9.8) | 3.9 (2.1-6.5) |
| Netherlands | 5.2 (2.8-8.4) | 3.5 (1.9-5.8) |
| Mexico | 10.9 (5.8-17.7) | 11.5 (6.4-18.3) |
| Iran | 11.4 (7.2-17.2) | 12.9 (8.4-18.8) |

Results are expressed as percentage and (95% confidence interval).
